# Modulating morphine-induced conditioned place preference: The role of diclofenac sodium

**DOI:** 10.1016/j.toxrep.2025.102174

**Published:** 2025-11-24

**Authors:** Haneen Amawi, Rawan Alhazaimeh, Alaa M. Hammad, Aseel O. Rataan, Sahar Alsheyab, Tayma Maklouf, Bahaa Al-Trad, Karem H. Alzoubi

**Affiliations:** a Department of Clinical Pharmacy and Pharmacy Practice, College of Pharmacy, Yarmouk University, Irbid 21163, Jordan; b Department of Biological Sciences, Faculty of Science, Yarmouk University, Irbid 21163, Jordan; c Department of Pharmacy, College of Pharmacy, Al-Zaytoonah University of Jordan, Amman, Jordan; d Department of Biomedical Sciences, College of Veterinary Medicine, King Faisal University, 31982 Al-Ahsa, Saudi Arabia; e Department of Pharmaceutical Sciences, College of Pharmacy, QU-Health, Qatar University, Doha, Qatar; f Department of Clinical Pharmacy, Faculty of Pharmacy, Jordan University of Science and Technology, Irbid 22110, Jordan

**Keywords:** Morphine, Diclofenac sodium, CPP model, Cox 1, Cox 2, NSAID

## Abstract

Morphine is known to induce strong reward-related behaviors, contributing to its high addiction potential. Non-steroidal anti-inflammatory drugs (NSAIDs), such as diclofenac sodium, have been suggested to modulate neuroinflammatory pathways involved in addiction. This study aimed to evaluate the effect of diclofenac sodium on morphine-induced conditioned place preference (CPP) in rats and investigate its underlying anti-inflammatory and antioxidant mechanisms. Female rats were subjected to a morphine-induced conditioned place preference (CPP) protocol. Diclofenac sodium (25 mg/kg) was administered 30 min prior to morphine conditioning sessions via injection. Post-conditioning, brain tissue samples were analyzed to measure the mRNA expression levels of cyclooxygenase enzymes (*Cox1*, *Cox2*), nuclear factor kappa B (*Nf-κB*), interleukin-6 (*Il-6*), and interleukin-1β (*Il-1β*). Oxidative stress in serum samples was assessed through catalase (CAT), superoxide dismutase (SOD), and myeloperoxidase (MPO) enzyme activity. Morphine significantly induced CPP, indicating a strong reward effect. Diclofenac sodium administration markedly attenuated this morphine-induced seeking behavior. This behavioral effect was accompanied by a significant reduction in the expression levels of *cox1*, *cox2*, *nf-κB*, and *il-6, and a significant increase in il-1β mRNA levels compared to the* morphine group. Additionally, diclofenac sodium significantly reduced oxidative stress, as indicated by decreased SOD activity when combined with morphine compared to the morphine group. In conclusion, Diclofenac sodium effectively attenuates morphine-induced reward behavior in the CPP model, potentially through modulation of inflammatory and oxidative stress pathways. These findings support the therapeutic potential of diclofenac sodium in managing opioid-seeking behaviors and provide insights into its anti-inflammatory and antioxidant mechanisms of action.

## Highlights

• Diclofenac sodium effectively attenuates morphine-induced reward behavior in the CPP model.
• This is potentially through modulation of inflammatory and oxidative stress pathways.
• These findings support the therapeutic potential of diclofenac sodium in managing opioid-seeking behaviors and provide insights into its anti-inflammatory and antioxidant mechanisms of action.

## 1 Introduction

Drug-seeking behavior is a persistent and relapsing condition, rooted in the prolonged effects of neurochemical effects of addictive substances [1]. Causal use can evolve into dependence and addiction through molecular changes that rewire the brain, creating a challenging environment for individuals trying to quit, despite awareness of harm [2]. Addiction may be substance-related (e.g., drugs, alcohol), or behavioral (e.g., gambling, cyber addiction), and follows a cyclical pattern influenced by neuroplasticity changes in the brain’s reward, stress, and executive function systems [3], [4]. It causes serious personal and social consequences, including financial instability, legal issues, and deteriorating mental health. Anxiety and depression are prevalent, too, with 40 % reporting suicidal thoughts [5]. Initiation often occurs at ages 17–18, with 16.9 % of secondary students reporting drug use, predominantly males [6]. Not all drug users develop addiction; genetic, environmental, and developmental factors determine susceptibility. Drug administration methods also affect the risk, with injectable drugs particularly are concerning [7]. Additionally, drug accessibility increases the likelihood of addiction [8]. Current pharmacotherapies may alleviate withdrawal symptoms and reduce cravings, but often lead to tolerance.

Morphine, although vital for pain management, can lead to tolerance, dependence, and abuse [9]. It triggers excessive dopamine release in the mesolimbic pathway, reinforcing drug use and diminishing responsiveness to natural rewards [10]. Moreover, chronic morphine reduces opioid receptor availability, increasing dose requirements [11], [12]. Other neurotransmitters, including glutamate, GABA, and serotonin, also contribute to craving, tolerance, and withdrawal symptoms.

An increasing body of evidence confirms that neuroinflammation plays a critical role in the persistence of addiction as well. Toll-like receptors (TLRs) activation, especially TLR2, increases inflammation and contributes to drug-seeking behavior [13]. Additionally, peripheral immune cells infiltrate the brain, further promoting inflammation [14]. The prefrontal cortex (PFC) regulates addiction through decision-making and emotional control via its connection to the nucleus accumbens (NAc) [15], [16], [17], [18]. By modulating NAc activity, the PFC helps regulate impulsive behavior and maintain goal-directed actions [19]. However, addictive substances disrupt this balance. Prolonged addictive drug use activates microglia and astrocytes, producing pro-inflammatory cytokines such as IL-1β, IL-6, and TNF-α, which further contribute to addiction [20]. Also, NF-κB is implicated in opioid addiction, making it a potential therapeutic target [21].

Thus, targeting inflammatory pathways using nonsteroidal anti-inflammatory drugs (NSAIDs) may offer a promising therapeutic avenue to modulate addiction. For example, diclofenac sodium, a COX inhibitor widely used for pain relief, has shown promising potential in attenuating opioid related addiction behavior [22], possibly by attenuating neuroinflammation that is induced by opioid withdrawal and drug-seeking behavior [23]. Therefore, in this study, we aim to assess the potential of diclofenac sodium in attenuating morphine-induced seeking behavior and explore the possible role of neuroinflammation.

The conditioned place preference (CPP) paradigm is a well-established behavioral model for assessing drug-seeking behavior and reward-associated learning, by pairing drug exposure with a specific environment; increased time spent in the drug-paired chamber reflects addiction-like motivational learning [24]. Thus, CPP is a suitable model to determine the potential role of diclofenac sodium in reversing morphine induced seeking behavior. Accordingly, investigating the morphine effects on neuroimmune pathways and inflammatory and oxidative markers is a relevant approach for studying the potential role of diclofenac sodium in reversing morphine-induced CPP.

COX-1/COX-2 regulates prostaglandin-driven neuroinflammation, and chronic opioid use upregulates COX-2 in addiction-related circuits [25]. IL-1β and IL-6 are key glial-derived cytokines that are elevated after opioid exposure, contributing to synaptic changes and reward learning [26]. NF-κB acts as a central regulator linking opioid-induced Toll-like receptor activation to cytokine expression and relapse vulnerability, especially in PFC, which is an important region critical for inhibitory control and decision-making [27]. Serum oxidative markers—including SOD, CAT, and MPO—reflect systemic oxidative imbalance associated with chronic opioid use and withdrawal [28]. These biomarkers together reflect neuroinflammation, glial activation, oxidative stress, and disruption of executive function underlying morphine-induced CPP. Therefore, this study aims to evaluate whether diclofenac sodium can attenuate morphine-induced drug-seeking behavior in rats using the CPP paradigm. It further examines COX-1, COX-2, IL-1β, IL-6, and NF-κB expression in the PFC, along with serum oxidative markers (SOD, MPO, and CAT), to elucidate the potential anti-inflammatory and antioxidant mechanisms underlying its therapeutic effects in addiction.

## 2 Materials and methods

### 2.1 Animals

All experimental procedures were approved by the Institutional Animal Care and Use Committee at Yarmouk University (Protocol # IACUC/2023/3). Thirty-two female Sprague-Dawley rats, weighing between 250 and 350 g, were obtained from the university's animal facility. They were housed under controlled conditions, maintaining temperature at 21–25°C, 55 % humidity, and a 12-hour light-dark cycle. The rats had free access to a standard chow diet and water.

### 2.2 Animal groups and treatments

**Fig. 1:**
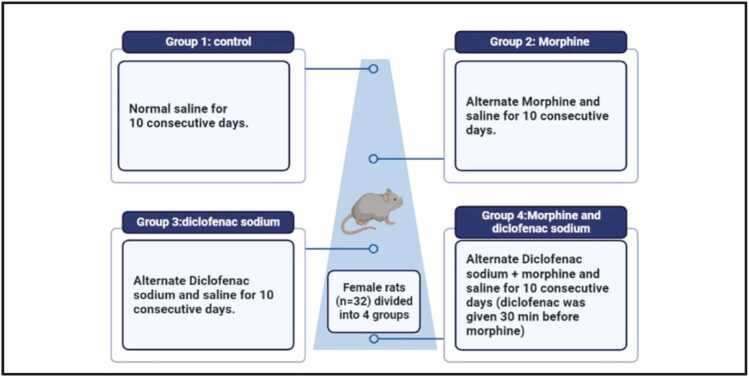
Schematic Diagram of the Study Design, the experimental groups: Saline (control) group received normal saline, while the Morphine group received alternate intraperitoneal (i.p.) injections of morphine/saline. The Diclofenac sodium group received alternate diclofenac sodium/saline injections, and the Morphine + Diclofenac sodium group received alternate doses of morphine and diclofenac sodium/saline injections. Each group received doses for 10 days.

The rats were assigned randomly to 4 different groups. Each experimental group consisted of eight rats. The Saline (control) group received normal saline, while the Morphine group received an intraperitoneal (i.p.) injection of 10 mg/kg morphine/saline. The diclofenac sodium group received a diclofenac sodium/saline injection (25 mg/kg), and the Morphine + diclofenac sodium group was given a morphine and diclofenac sodium/saline injection. By the end of the experiment, the rats were euthanized, and their brains were immediately extracted and stored at −80°C for further analysis (Fig. 1**).** Morphine and diclofenac sodium were purchased from Hikma Pharmaceuticals, Amman, Jordan. Morphine was dissolved in normal saline and administered intraperitoneally (i.p.) via injection. Similarly, Diclofenac sodium was extracted from ampules and injected intraperitoneally (i.p.).

### 2.3 Experimental design

The rats were involved in a preconditioning phase and a conditioning phase using the conditioning place preference (CPP) apparatus, which consists of a rectangular plexiglass chamber measuring 35 cm (width) × 35 cm (length) × 50 cm (height). Initially, the rat was placed in a neutral, exterior, colorless chamber. The apparatus was divided into two compartments—one white and one black—separated by a movable guillotine door. The white compartment featured vertical black and white stripes on a smooth white floor, while the black compartment had horizontal black and white stripes on a rough black floor. To track the rats’ movement between the compartments, an 18 W table light was positioned approximately 74 cm above the black compartment. A video camera was mounted above the apparatus to record the time spent in each compartment. The recorded footage was manually analyzed on a computer to assess the behavioral responses.

The preconditioning phase was conducted to ensure a non-biased design and lasted for 3 days to allow the rats to adapt to the apparatus surroundings. For the initial two days, a process called habituation took place, during which the rats were placed in both chambers for 30 min without any intervention. They were introduced at the entrance, the divider was removed, and they were allowed to explore freely. On the 3rd day, the rats' movements were recorded for 20 min using a video camera mounted above the apparatus. Between trials, the box was cleaned with normal saline to prevent scent cues from influencing behavior. The time spent in each chamber, either black or white, was manually calculated. To maintain a balanced and unbiased experimental design, rats were then randomly assigned to chambers, ensuring a similar average time spent in each. After that, for the morphine group, the conditioning stage was started by randomly assigning morphine to one compartment and saline to the other. Injections were administered alternately over a 10-day period, with each rat receiving morphine (10 mg/kg) on one day and saline on the next, resulting in a total of five pairings for each condition. On days 4, 6, 8, 10, and 12, rats received a morphine injection and were then placed in a single chamber for 30 min. On days 5, 7, 9, 11, and 13, they were given saline and confined to the corresponding chamber for the same duration. The compartment location (left or right) was also alternated, following the approach used in a previous study [29]

**Fig. 2:**
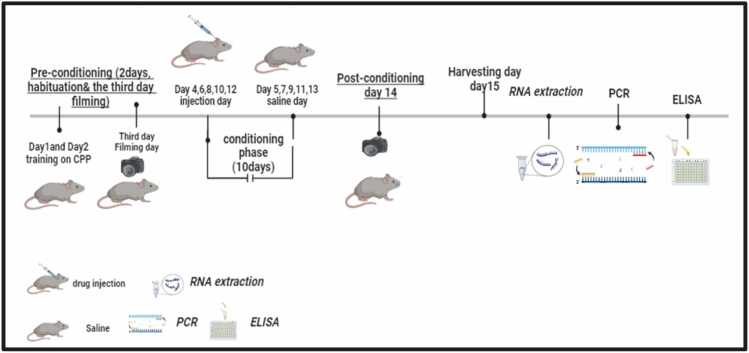
Timeline & Experimental protocol: preconditioning for 2 days with video filming on day 3, followed by conditioning stage from day 4 through day 13, and finally post conditioning at day 14. The rats were then euthanized, and their brains were collected for further analysis (PCR & ELISA).

In the morphine + diclofenac group, diclofenac sodium (25 mg/kg/day) was given 30 min before the morphine injection to allow sufficient distribution within the central nervous system before drug administration. On the 14th day, following the conditioning phase, the movement of each rat was recorded individually with the partition between the two compartments removed. A video camera mounted above the apparatus captured the time spent in each compartment over a 20-minute period. After completing the final behavioral test, the animals were euthanized with 100 mg/kg, i.p. of sodium pentobarbital, followed by decapitation, using a guillotine. The brains were collected immediately and stored at −80 °C. After that, the brains were sectioned by a cryostat device (CM1850, Leica, Germany) at −20 °C, and according to the rat brain atlas, the PFC regions were sectioned. Blood samples were collected from the neck immediately after decapitation into plain tubes and allowed to clot for 15 min. The samples were then centrifuged at 1000–2000 × g for 10 min, and the obtained serum was stored immediately at −80 °C in aliquots for further analysis **(**Fig. 2**).**

### 2.4 Gene expression analysis

Total RNA was isolated from the PFC using the QIAGEN RNA Purification Kit (Cat. #74104). Then it was transcribed to cDNA using EasyScript First-Strand cDNA Synthesis Super Mix (Code: AE301–02), as directed by the manufacturer. The reaction then included 10 μL of 2X BlasTaqTM qPCR MasterMix, 0.5 L each of forward and reverse primers, 2 μL of cDNA template, specific primers for the genes of interest as given in Table 1, and nuclease-free water to a final volume of 20 μL. The reaction conditions were set as follows: an initial denaturation at 95 °C for 3 min, followed by 40 cycles of 95 °C for 30 s and 60 °C for 1 min, using the Tianlong Real-Time PCR Detection System (Gentier48E). For normalization, glyceraldehyde 3-phosphate dehydrogenase (GAPDH) was used as a reference gene. The primers for COX1 and COX2 were designed using the NCBI BLAST software. Relative quantification was performed using the Bio-Rad CFX Maestro™ software with the ∆∆Ct method, and results were calculated using the 2 −ΔΔCt method.

**Table 1:** Primer sequence for the genes of interest.

Gene primer*	Sequences
il-1β forward il-1β reverse	5’-CAGCTCATATGGGTCCGACA −3’ 5’-CTGTGTCTTTCCCGTGGACC −3’
il-6- forward il-6-reverse	5’-TCCTACCCCAACTTCCAATGCTC-3’ 5’-TTGGATGGTCTTGGTCCTTAGCC-3’
nf-κB- forward nf-κB-reverse	5'-AAAAACGCATCCCAAGGTGC-3′ 5'-AAGCTCAAGCCACCATACCC-3′
cox1- forward cox1-reverse	5’-TGGTGGGGGTAGGAACTTTGACT-3’ 5’-GGCCATCTCCTTCTCTCCTGTG-3’
cox2-forward cox2-reverse	5’-AGACAGATTGCTGGCCGGGTTG-3’ 5’-TTCAGGGAGAAGCGTTTGCGG-3’
gapdh-forward gapdh-reverse	5’-CCCCCAATGTATCCGTTGTG-3’ 5’-TAGCCCAGGATGCCCTTTAGT-3’

* Abbreviations: il-1β; interleukin-1beta, il-6; interleukin 6, nf-κB; Nuclear factor kappa-light-chain-enhancer of activated B cells, cox1; cyclooxygenase 1, cox 2; cyclooxygenase 2.

### 2.5 Antioxidant enzyme activity assay

All three assays (CAT, SOD, & MPO enzyme assays) were performed according to the manufacturer's instructions. CAT, SOD, and MPO antioxidant enzyme activity have been measured in the serum of the treated rats using a commercially available activity assay kit (Sunlong Biotech Co., China). For the CAT activity, the wavelength was adjusted to 240 nm with a blank. The CAT working reagent was incubated in a 37 ℃ water bath for 10 min. Subsequently, 190 μL of the CAT working reagent and 10 μL of the sample were added to a 96-well UV flat-bottom plate. Immediately after mixing, the absorbance was measured at 240 nm at the initial time (A1) and again after 1 min (A2). The CAT activity was calculated using the following equation:

ΔA=A1-A2. For SOD activity, it was measured in a similar way, with the wavelength set to 560 nm. Serum MPO levels were also measured. A 50 μL sample was pipetted into a 96-well plate, with a blank well serving as a control. Each sample received 40 μL of dilution solution, mixed gently, and incubated at 37 °C for 30 min. Next, 50 μL of HRP-conjugated reagent was added (except in the control well) and incubated for another 30 min at 37 °C. Subsequently, 50 μL each of chromogen solutions A and B were added, gently mixed, and incubated for 15 min at 37 °C. The reaction was stopped by adding 50 μL of stop solution, and the absorbance was measured.

### 2.6 Statistical analysis

The time spent in the conditioning chambers following each phase of CPP was examined using two-way repeated measures ANOVA (Time treatment) and Sidak's multiple comparisons test. The real-time PCR results of *cox1, cox2, nf-κB, il-1β, il-6*, and the ELISA kit result of MPO, SOD, and CAT enzyme activity were analyzed using one-way ANOVA followed by Tukey’s multiple comparisons test. All data were analyzed using GraphPad Prism, with significance defined as p < 0.05.

## 3 Result

### 3.1 Behavioral results

#### 3.1.1 Effects of saline, morphine, and the combination of morphine + diclofenac on conditioned place preference in rats

**Fig. 3:**
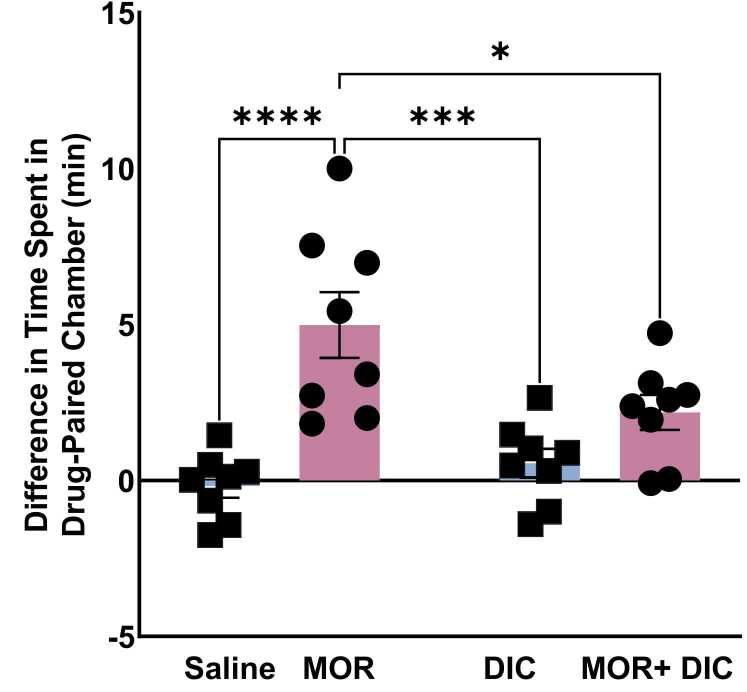
The Difference in Time Spent in Drug–Paired Chamber (min). DIC; Diclofenac, MOR; Morphine. Data are represented as mean ± SEM. (*: p < 0.05, **: p < 0.01, ***: p < 0.001, ****: p < 0.0001, n = 8 for each group) blue: without Morphine Treatment, pink: with Morphine Treatment.

Analysis of the difference in time spent in the drug-paired chamber revealed a significant effect of treatment (Fig. 3). Rats in morphine group, received 10 mg/kg morphine i.p., 5 doses, exhibited a significant increase in time spent in the drug-paired chamber compared to saline, diclofenac, and the morphine + diclofenac groups (p value < 0.0001, 0.001, and 0.05 respectively). A Two-way ANOVA followed by Tukey’s multiple comparisons showed a significant main effect of Morphine [F(1,28)= 25.98,p < 0.0001], a significant main effect of Diclofenac [F(1,28)= 24.02,p < 0.0001] and a significant main effect of interaction Morphine X Diclofenac [F(1,28)= 7.03,p = 0.013] indicating that morphine induced a strong conditioned place preference (CPP) compared to all other groups. Notably, the combination of morphine with diclofenac attenuated this preference where rats spent less time in drug paired chamber compared to morphine group. Further, no significant difference in time spent in the chambers was detected between saline, diclofenac, and morphine+ diclofenac groups, as seen in Fig. 3.

### 3.2 Molecular results

#### 3.2.1 Effects of morphine and diclofenac sodium on *cox1* and *cox2* mRNA expression in the PFC region

**Fig. 4:**
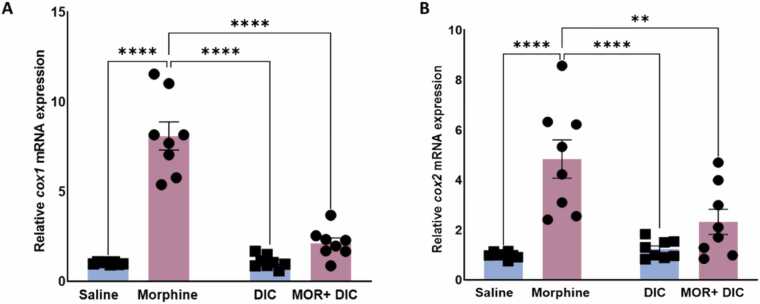
Effects of saline, Morphine (10 mg/kg), Diclofenac (25 mg/kg), and Morphine + Diclofenac on the relative expression of mRNA of (A) *Cox1*. (B) *cox2* Data are represented as mean ± SEM. **: p < 0.01, ****: p < 0.0001, n = 8 for each group). blue: without Morphine Treatment, pink: with Morphine Treatment.

Analysis of *cox1* mRNA expression in PFC showed a significant modulation by treatment, Fig. 4**A**. Rats in the morphine group (10 mg/kg morphine i.p., 5 doses) demonstrated a significant upregulation of *cox1* mRNA expression compared to the saline, diclofenac (25 mg/kg), and morphine + diclofenac groups. A Two-Way ANOVA followed by Tukey’s multiple comparisons revealed a significant effect of Morphine [F(1,28)= 55.98,p < 0.0001], a significant main effect of Diclofenac [F(1,28)= 48.75,p < 0.0001] and a significant main effect of interaction Morphine X Diclofenac [F(1,28)= 50.92,p = 0.013], indicating that morphine significantly increased *cox1* mRNA expression. Diclofenac, on the other hand, reversed this elevation when combined with morphine in the morphine+diclofenac group.

Analysis of *cox2* mRNA expression in PFC showed a significant modulation by treatment, Fig. 4**B**. Rats in the morphine group (10 mg/kg morphine i.p., 5 doses) demonstrated a significant upregulation of *cox2* mRNA expression compared to the saline, diclofenac (25 mg/kg), and morphine + diclofenac groups. A Two-Way ANOVA followed by Tukey’s multiple comparisons revealed a significant effect of Morphine [F(1,28)= 55.98,p < 0.0001], a significant main effect of Diclofenac [F(1,28)= 48.75,p < 0.0001] and a significant main effect of interaction Morphine X Diclofenac [F(1,28)= 50.92,p = 0.013], indicating that morphine significantly increased *cox2* mRNA expression. Diclofenac, on the other hand, reversed this elevation when combined with morphine in the morphine+diclofenac group.

### 3.3 Effects of morphine and diclofenac sodium on *il-6*, *il-1β*, and *nf-kb* mRNA expression in the PFC region

**Fig. 5:**
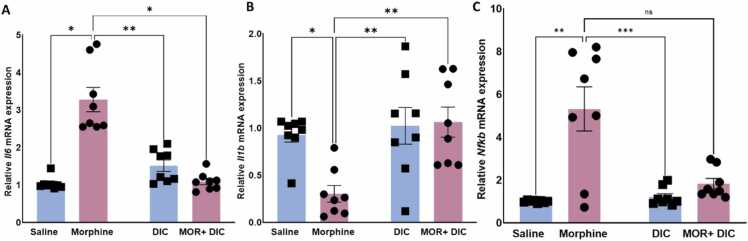
Effects of saline, Morphine (10 mg/kg), Diclofenac (25 mg/kg), and Morphine + Diclofenac on the relative expression of mRNA of (A) *Il6*, (B) Il1b, and (C) Nfkb. Data are represented as mean ± SEM. **: p < 0.01, ****: p < 0.0001, n = 8 for each group). blue: without Morphine Treatment, pink: with Morphine Treatment.

Analysis of *il-6* mRNA expression in PFC showed a significant modulation by treatment, Fig. 5**A**. Rats in morphine group (10 mg/kg morphine i.p., 5 doses) demonstrated a significant upregulation of *il-6* mRNA expression compared to the saline, diclofenac (25 mg/kg), and morphine + diclofenac groups. A Two-Way ANOVA followed by Tukey’s multiple comparisons revealed a significant effect of Morphine [F(1,28)= 7.694,p = 0.0096], a significant main effect of Diclofenac [F(1,28)= 8.690,p = 0.0063] and a significant main effect of interaction Morphine X Diclofenac [F(1,28)= 4.478,p = 0.0341], indicating that morphine significantly increased *il-6* mRNA expression. Diclofenac, on the other hand, reversed this elevation when combined to morphine in morphine+ diclofenac group.

Similarly, analysis of *il-1β* mRNA expression in PFC showed a significant modulation by treatment, Fig. 5B. Rats in morphine group (10 mg/kg morphine i.p., 5 doses) demonstrated a significant downregulation of *il-1β* mRNA expression compared to the saline, diclofenac (25 mg/kg), and morphine + diclofenac groups. A Two-Way ANOVA followed by Tukey’s multiple comparisons revealed a significant effect of Morphine [F(1,28)= 5.733,p = 0.0236], a significant main effect of Diclofenac [F(1,28)= 9.481,p = 0.0046] and a significant main effect of interaction Morphine X Diclofenac [F(1,28)= 4.446,p = 0.0441], indicating that morphine significantly decreased *il-1β* mRNA expression. Diclofenac reversed this decrease when combined with morphine in the morphine+diclofenac group.

Finally, analysis of *nf-kb* mRNA expression in PFC showed a significant modulation by treatment, Fig. 5C. Rats in morphine group (10 mg/kg morphine i.p., 5 doses) demonstrated a significant upregulation of *nf-kb* mRNA expression compared to the saline, diclofenac (25 mg/kg), and morphine + diclofenac groups. A Two-Way ANOVA followed by Tukey’s multiple comparisons revealed a significant effect of Morphine [F(1,28)= 6.767,p = 0.0145], a significant main effect of Diclofenac [F(1,28)= 15.345,p = 0.0005] and a significant main effect of interaction Morphine X Diclofenac [F(1,28)= 28.654,p < 0.0001], indicating that morphine significantly increased *nf-kb* mRNA expression. Diclofenac reversed this elevation when combined with morphine in the morphine+diclofenac group.

### 3.4 Effects of morphine and diclofenac sodium on serum antioxidant enzyme activity

**Fig. 6:**
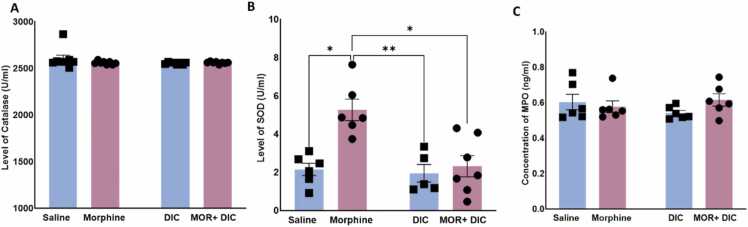
Effects of saline, Morphine (10 mg/kg), Diclofenac (25 mg/kg), and Morphine + Diclofenac on the antioxidant enzyme activity of (A) Catalase (CAT), (B) SOD, (C) MPO. Data are represented as mean ± SEM. **: p < 0.01, ****: p < 0.0001, n = 8 for each group). blue: without Morphine Treatment, pink: with Morphine Treatment.

Analysis of level of CAT in PFC showed a non-significant modulation by treatment, Fig. 6A. A Two-Way ANOVA followed by Tukey’s multiple comparisons revealed a non-significant effect of Morphine [F(1,28)= 1.245,p = ns], a non-significant main effect of Diclofenac [F(1,28)= 0.665,p = ns] and a non-significant main effect of interaction Morphine X Diclofenac [F(1,28)= 0.8127,p = ns], indicating that morphine did not affect CAT activity in PFC.

In contrast, analysis of SOD activity in PFC showed a significant modulation by treatment, Fig. 6B. Rats in morphine group (10 mg/kg morphine i.p., 5 doses) demonstrated a significant increase in SOD activity compared to the saline, diclofenac (25 mg/kg), and morphine + diclofenac groups. A Two-Way ANOVA followed by Tukey’s multiple comparisons revealed a significant effect of Morphine [F(1,28)= 8.723,p = 0.0069], a significant main effect of Diclofenac [F(1,28)= 8.771,p = 0.0074] and a significant main effect of interaction Morphine X Diclofenac [F(1,28)= 9.441,p = 0.0058], indicating that morphine significantly increased SOD activity. While diclofenac reversed this elevation when combined to morphine in morphine+ diclofenac group.

Finally, Analysis of the level of MPO in PFC showed a non-significant modulation by treatment, Fig. 6C. A Two-Way ANOVA followed by Tukey’s multiple comparisons revealed a non-significant effect of Morphine [F(1,28)= 0.987,p = ns], a non-significant main effect of Diclofenac [F(1,28)= 1.345,p = ns] and a non-significant main effect of interaction Morphine X Diclofenac [F(1,28)= 1.15643,p = ns], indicating that morphine did not affect MPO activity in PFC.

## 4 Discussion

Morphine is the primary opiate alkaloid, possessing strong addictive qualities. Although it is a powerful analgesic, its usage is limited because of tolerance, dependence, and the danger of misuse [30], [31]. Craving, anxiety, irritability, sweating, dysphoria, lacrimation, runny nose, abdominal pain, and diarrhea are some of the signs of opiates withdrawal in humans [32]. In this study, morphine administration to rats at a dose of 10 mg/kg resulted in significant induction of seeking behavior using the CPP behavior model. This result is consistent with several previous animal studies. For example, Arani et al. confirmed that CPP was strongly induced by the treatment with a dose of 10 mg/kg morphine, which resulted in a longer time spent by rats in the morphine chamber than the saline chamber [31]. Another study conducted by McKendrick et al. demonstrated that the mice that were conditioned with 10 mg/kg morphine intraperitoneally for 5 days spent significantly less time in the open arm of the elevated plus-maze after morphine conditioning and had strong morphine CPP on CPP test day [33]. On the other hand, diclofenac sodium in our study, administered to rats as a 30-min pretreatment, followed by morphine (10 mg/kg) for ten days (5 cycles), reversed the morphine induced seeking behavior in CPP model.

The cyclooxygenase (COX) enzymes (COX1 and COX2) are involved in the production of prostaglandins (PGs) in the human body [34]. In our study, morphine resulted in significantly increased mRNA expression levels of *cox1* and *cox2* in the rat brains, particularly in the PFC region. These results may support the role of the COX pathway and prostaglandins in the morphine-induced CPP. Consistent with these results, it was previously reported that alcohol consumption increases the production of PGs, and blocking PG production attenuates the behavioral effects of alcohol. In addition, PG has been associated with cocaine abuse [34], [35]. Thus, morphine-induced CPP can be attributed, at least in part, to the increase in prostaglandin production through COX enzymes.

Interestingly, in this study, diclofenac sodium was able to reverse the morphine induced upregulation in mRNA expression levels of *cox1* and *cox2* in the PFC region*.* It is well known that diclofenac is a strong COX inhibitor. For example, Yilmaz et al. confirmed that diclofenac significantly reduced the expression of COX-2 [36]. Thus, reversing behavioral findings could be partially explained by the diclofenac effect as a COX inhibitor, where a previous study showed that COX pathway inhibition can reduce morphine-induced conditioned place preference (CPP) [22]. Diclofenac sodium can modulate the PGs by inhibiting COX enzymes, which are involved in PG production [37]. Another study conducted on male Sprague-Dawley rats (weighing 250–350 g) investigated the effects of diclofenac following focal penetrating traumatic brain injury (TBI). The rats were randomly assigned to receive diclofenac (5 μg intralesional) immediately after TBI. Researchers found that diclofenac sodium reduced COX-2 levels and was associated with decreased apoptosis (programmed cell death) and a smaller lesion area in the brain following severe injury, highlighting its anti-inflammatory properties in the CNS [38]. Additionally, a recent study reported that cigarette smoke exposure increased the amygdala and hippocampus tissue content of dopamine, serotonin, glutamate, glutamine, and GABA, and treatment with aspirin, another COX inhibitor, normalized this effect [39]. All these studies may indicate that COX enzymes have a potential role in modulating drug abuse and reward mechanisms.

COX enzymes are involved in the production of pro-inflammatory PGs. Neuroinflammation has been linked to several neurological disorders, including addiction [34]. Chronic drug use can result in neuroinflammation, and the COX may play a role in this process. Our study supported the hypothesis that the inhibition of the COX cascade might participate in the modulation of the reward pathways by modulating neuroinflammation [22]. The release of proinflammatory cytokines in the CNS, such as IL-6, TNF-α, NF-κB, and IL-1β, is increased in opioid addiction in response to neuroinflammation [40]. Our results showed that rats with morphine-induced seeking behavior expressed higher levels of *il-6* and *nf-kb*. These findings are consistent with previous reports. For example, elevated PFC's pro-inflammatory cytokines (NF-κB and IL-6) may contribute to anxiety-like behavior following repeated morphine administration (10 mg/kg for BID for eight days) [41]. Another study showed that mice that received two daily injections of morphine sulfate (10 mg/kg) for seven days had significantly elevated levels of IL-6, which is also consistent with our findings [42]. On the other hand, our study found that diclofenac sodium significantly reversed the morphine effects on the relative mRNA expression levels of *nf-kb* and *il-6* in the PFC region of the rat brain. This result is consistent with a previous study where diclofenac sodium showed anti-inflammatory effects through the modulation in the TLR4/NF-κB signaling pathway, and resulted in a significant decrease in *nf-kb* levels [43].

Interestingly, morphine in our study induced a significant reduction in *il-1β* mRNA expression in the PFC region, which could be explained by morphine-induced inflammation in later phases. This finding is consistent with what has been previously confirmed during the late phase of both the resolving and persistent inflammation models: the rate of synthesis of IL-1 family molecules is usually decreased [44]. In addition, acute and chronic morphine use induce a differential expression of cytokines in the brain. Chronic morphine administration elicited a decrease in IL-1β in the frontal cortex but produced no changes in other brain areas (striatum, hippocampus, and cerebellum) [45]. Most studies have reported decreased IL-1β levels when NSAIDs are administered, as these anti-inflammatory drugs reduce proinflammatory cytokines in CNS. A previous study found that IL-1β levels decreased after treatment with celecoxib and ibuprofen following traumatic brain injury (TBI) in male rats [46]. However, our findings showed that diclofenac sodium reverses morphine induced reduction of *il-1β*. This suggests that certain NSAIDs, including diclofenac sodium, may trigger pro-inflammatory responses under specific conditions, particularly during the late phase of neuroinflammation. The observed increase in *il-1β* levels may be attributed to the drug’s modulation of immune responses, possibly through the activation of specific signaling pathways. Further research is needed to understand these findings.

A growing body of evidence has indicated that oxidative stress is involved in the development of addiction to several addictive drugs, including morphine [47]. Several previous studies indicated that after morphine exposure, the activity of SOD and CAT enzymes was decreased [47], [48], Additionally, MPO has been linked to neurological diseases, with significantly higher peripheral blood concentrations observed in Alzheimer's disease patients [49]. This elevation may be attributed to MPO secretion by activated neutrophils and macrophages at sites of inflammation [50]. NSAIDs at therapeutic doses have been shown to modulate the oxidative burst of neutrophils, potentially providing benefits in inflammatory conditions characterized by excessive ROS production [51]. However, in our current study, only SOD activity was significantly increased in the morphine-treated group, while CAT and MPO activities remained unchanged between different treatment groups. The increased SOD levels in the morphine-treated group may be attributed to compensatory mechanisms aimed at counteracting oxidative stress. This elevation in SOD could be linked to a hyperdopaminergic state in the nucleus accumbens, caudate nucleus, and amygdala, resulting in increased production of superoxide and H₂O₂ radicals [52]. However, treatment with diclofenac sodium reversed this increase, likely due to the antioxidant properties of NSAIDs, which can modulate SOD activity.

Regarding CAT enzyme activity, previous studies have suggested that chronic morphine exposure triggers neuroinflammation, contributing to oxidative stress and potentially reducing CAT activity [47]. Another study investigating the effects of ibuprofen (10, 20, and 30 mg/kg of ibuprofen for 21 days) found that CAT activity increased due to activating anti-inflammatory pathways [53]. However, as mentioned above, no significant differences in CAT activities were observed between treatment groups in our study. Similar to CAT, no significant differences in MPO levels were detected between our treatment groups. We attribute these discrepancies to the fact that our measurements were conducted on serum rather than specific brain regions. Therefore, we suggest future studies focus on assessing CAT and MPO within distinct brain regions.

This study has several limitations; one limitation is the absence of locomotor and sickness control assessments (e.g., open field or rotarod tests) to exclude the possibility that diclofenac’s effect on CPP reflects sedation or malaise rather than specific attenuation of morphine reward. Future studies will include these controls to confirm that the observed behavioral changes are not due to alterations in motor activity or general health status. Another limitation is that oxidative stress was assessed only in serum, providing systemic but not region-specific information. Since CPP relies on CNS reward circuitry, future studies will include brain region–specific measurements to better relate behavioral changes to central oxidative and inflammatory mechanisms. Further, the absence of histopathological evaluation in this study is also a limitation. It would have provided valuable confirmation of tissue-level changes corresponding to the observed molecular alterations; future studies are planned to include such analyses. Another limitation of this study is the use of a single diclofenac dose, which precludes evaluation of potential dose-dependent effects. The selected dose was based on prior studies showing behavioral efficacy without toxicity; however, future work should include a dose–response design to more fully characterize diclofenac’s influence on morphine-associated behaviors and molecular outcomes. This study did not assess neurotransmitter levels or signaling pathways directly. Future studies will include analyses of dopaminergic and serotonergic turnover and receptor activity (e.g., D1/D2, 5-HT1A/5-HT2A) in reward-related brain regions such as the nucleus accumbens, amygdala, and prefrontal cortex to further elucidate the neurochemical mechanisms underlying diclofenac’s effects on morphine dependence.

## 5 Conclusion

In conclusion, our findings show that morphine induced seeking behavior in rats using the CPP. Also, we showed for the first time that injection administration of diclofenac sodium (25 mg/kg) can attenuate morphine induced seeking behavior in rats using a CPP paradigm. Further, the attenuation in morphine induced seeking behavior by diclofenac was associated with significant reduction in relative mRNA levels of *cox 1, cox2, nf-κB, il-6*, and a significant increase in *il-1β*. Further, diclofenac sodium significantly reduced oxidative stress markers when combined with morphine. These data reveal that diclofenac sodium has potential in modulating morphine-induced seeking behaviors, and that, in part, this may be due to its modulation of key neuroinflammation mechanisms.

## Ethics approval and consent to participate

The study protocol was approved by the Institutional Animal Care and Use Committee (IACUC) at Yarmouk University, Irbid, Jordan.

## Human and animal rights

The study protocol was approved by the Institutional Animal Care and Use Committee at Yarmouk University, Irbid, Jordan.

## Consent for publication

Not applicable.

## Funding

This project was supported by a grant from the Deanship of Research, Yarmouk University, Irbid, Jordan.

## CRediT authorship contribution statement

**Alzoubi Karem:** Writing – review & editing, Validation, Supervision, Resources, Project administration, Investigation, Data curation, Conceptualization. **Bahaa Al-Trad:** Writing – original draft, Visualization, Validation, Supervision, Resources, Project administration, Investigation, Funding acquisition, Data curation, Conceptualization. **Tayma Maklouf:** Writing – original draft, Visualization, Methodology, Investigation, Formal analysis, Data curation, Conceptualization. **Haneen Amawi:** Writing – review & editing, Writing – original draft, Validation, Supervision, Resources, Project administration, Methodology, Investigation, Funding acquisition, Formal analysis, Data curation, Conceptualization. **Sahar Alsheyab:** Writing – original draft, Visualization, Methodology, Investigation, Data curation, Conceptualization. **Aseel O. Rataan:** Writing – original draft, Visualization, Supervision, Software, Resources, Project administration, Investigation, Data curation, Conceptualization. **Alaa M. Hammad:** Writing – original draft, Validation, Supervision, Resources, Project administration, Investigation, Data curation, Conceptualization. **Rawan Alhazaimeh:** Writing – original draft, Visualization, Methodology, Investigation, Formal analysis, Data curation, Conceptualization.

## Declaration of Competing Interest

The authors declare that they have no known competing financial interests or personal relationships that could have appeared to influence the work reported in this paper.

## Data Availability

Data will be made available on request.
